# The Adsorption Process and Mechanism of Benzo[a]pyrene in Agricultural Soil Mediated by Microplastics

**DOI:** 10.3390/toxics12120922

**Published:** 2024-12-19

**Authors:** Zhengyi Zhu, Lijuan Sun, Qin Qin, Yafei Sun, Shiyan Yang, Jun Wang, Yang Yang, Guangkuo Gao, Yong Xue

**Affiliations:** 1 Eco-Environment Protection Research Institute, Shanghai Academy of Agricultural Sciences, Shanghai 201403, China; magiczzy2@163.com (Z.Z.); sunliuliu2012@126.com (L.S.);; 2 College of Marine Ecology and Environment, Shanghai Ocean University, Shanghai 201306, China; 3 Key Laboratory of Low-Carbon Green Agriculture in Southeastern China, Ministry of Agriculture and Rural Affairs, Shanghai 201403, China

**Keywords:** soil, microplastics, benzo[a]pyrene, adsorption, migration

## Abstract

The coexistence of microplastics and benzo[a]pyrene (BaP) in the environment, and their interactions within agricultural soils in particular, have garnered widespread attention. This study focused on the early-stage interactions between microplastics and BaP, aiming to uncover their initial adsorption mechanisms. Despite the significant environmental toxicity of both pollutants, research on their mutual interactions in soil is still limited. This study conducted adsorption thermodynamics and kinetics experiments to explore the effects and mechanisms of various microplastics (polyethylene (PE), polystyrene (PS), and polyvinyl chloride (PVC)) on the adsorption of BaP. Using advanced techniques such as scanning electron microscopy (SEM), X-ray photoelectron spectroscopy (XPS), and Fourier transform infrared (FTIR) spectroscopy, this study explored the surface characteristics of microplastics and their interactions with BaP. The results demonstrated that PVC microplastics exhibited the highest adsorption capacity for BaP, which was primarily due to π–π interactions and increased hydrophobicity. In the soil–microplastic blend systems, BaP was predominantly found on microplastics, enhancing the soil’s adsorption capacity for BaP, particularly PVC, which showed an adsorption capacity 3.69 times greater than that of soil alone. Density functional theory (DFT) simulation calculations indicated that the binding energy of BaP for PVC pretreated with soil was −59.16 kJ/mol, whereas it was −53.02 kJ/mol for untreated PVC, −39.35 kJ/mol for PE, and −48.84 kJ/mol for PS. These findings suggest that soil pretreatment enhances the adsorption stability of PVC for BaP, further elucidating the potential mechanisms behind the increased adsorption capacity in the soil–microplastic system. These findings confirm that microplastics serve as effective vectors for organic pollutants such as BaP, significantly influencing their environmental behavior in soils, and provide essential theoretical support for assessing the environmental toxicity and migration behaviors of microplastics and associated organic contaminants.

## 1. Introduction

Microplastics, as emerging environmental pollutants, have garnered widespread attention globally due to their persistent presence and ecological impact [1]. Defined as plastic particles smaller than 5 mm [2], microplastics have been detected in diverse environments, including marine [3], soil [4], and even polar regions [5]. Their origins are multifaceted, stemming from sources such as synthetic textile fibers released during washing, tire wear, and the breakdown of larger plastic debris in both industrial and domestic contexts [6,7]. Moreover, out of a large volume of plastic waste, only 9% is recycled, 12% is incinerated, and the remaining 79% is buried in soils [8].

Farmland soils, critical for global food production, are among the environments most severely impacted by microplastics, with contamination levels reaching 78 ± 13 particles/kg in some regions [9]. This contamination not only threatens soil health but also increases the mobility and persistence of hazardous pollutants like polycyclic aromatic hydrocarbons (PAHs) [10,11]. Microplastics have demonstrated significant adsorption properties for organic pollutants, acting as carriers that can enrich and transport PAHs due to the natural tendency of lipophilic compounds to adhere to plastic surfaces. The adsorption process involves both physical adsorption, influenced by surface area, and chemical adsorption, driven by hydrophobic interactions [12,13]. Physical adsorption occurs through van der Waals forces, while the strong hydrophobic nature of BaP facilitates its transfer from the aqueous phase to the hydrophobic surfaces of microplastics such as polyethylene (PE) and polystyrene (PS). Chemical adsorption may involve π–π interactions or hydrogen bonding between polar groups on microplastic surfaces and BaP molecules [14]. Research has shown that microplastics such as PE, PS, and polyvinyl chloride (PVC) exhibit stronger adsorption capabilities for PAHs than natural sediment particles, posing significant environmental risks due to their propensity to accumulate smaller particles [14]. In addition to PE, PS, and PVC, other commonly found microplastics include polypropylene (PP) and polyamide (PA), which are widely used in packaging and textiles, and have been reported to interact with organic pollutants in soil environments [15,16]. The affinity of nonpolar organic compounds for microplastics is positively correlated with their hydrophobicity, while the adsorption of polar organic compounds is more susceptible to environmental conditions (e.g., temperature and pH) [17]. After soil aging, the surface properties of microplastics change, especially when hydrophobicity decreases, surface area increases, and oxygen-containing functional groups increase in abundance, significantly increasing their adsorption capacity for organic pollutants and thereby changing the distribution of pollutants in the soil [18]. These different results indicate that the adsorption capacity of different microplastics for organic pollutants may vary [19].

Benzo[a]pyrene (BaP), a five-ring PAH, is known for its high toxicity and carcinogenic potential, making it one of the most hazardous PAHs. This substance is difficult to degrade in the natural environment, and its abundance is often closely related to that of other PAH components, making it a useful marker for assessing the level of PAH pollution in the environment. BaP primarily enters soils through landfill waste, sewage irrigation, vehicle emissions, industrial discharge, household waste, and agricultural activities [20]. Since microplastics enter farmland soils through agricultural films, it is highly likely that both pollutants coexist in the soil, especially in farmland soils along highways. Microplastics have a large specific surface area and highly hydrophobic surfaces, which can increase the mobility of BaP in soil through adsorption, making it more easily mobile in soil. This study focused on the early-stage interactions between microplastics and BaP to uncover the initial adsorption mechanisms. This interaction may lead to the easier movement of BaP in soil, increasing the possibility of it entering groundwater and thereby increasing environmental and human health risks. Therefore, the presence of microplastics in soil systems may influence the fate of BaP in agricultural systems. In this study, different types of soil microplastics were investigated to analyze the effects of microplastics on the adsorption of BaP and the interaction mechanism and to elucidate the distribution of BaP in the soil–microplastic system.

## 2. Materials and Methods

### 2.1. Materials

Polyethylene (PE), polystyrene (PS), and polyvinyl chloride (PVC) with a particle size of 100 µm were purchased from the Guangdong Hengfa Plasticization Factory, Guangzhou, China. These three types of microplastics were selected because they are widely present in soil [17].

BaP (purity > 96%) was purchased from China National Pharmaceutical Group Chemical Reagent Co., Ltd., Shanghai, China. A certain amount of the BaP monomer standard was weighed using a microbalance and dissolved in acetone to prepare a BaP acetone stock solution with a target concentration of 4 g L^−1^, which was sealed and stored at −4 °C for later use.

The experimental soil was collected from the top layer (0~20 cm) of an open-air vegetable field at the Shanghai Academy of Agricultural Sciences Experimental Station in Zhuanghang Town, Fengxian District, Shanghai (30°53′39″ N, 121°23′47″ E). After the removal of gravel, plant roots, and debris residues, the natural structure of the experimental soil was lightly broken into blocks with a diameter of approximately 1 cm and then air-dried naturally. The soil was sieved through a 10-mesh sieve for subsequent physicochemical property determination and adsorption experiments. The basic properties of the soil were measured and are as follows: pH of 8.40, organic matter content of 18.31 g kg^−1^, alkali hydrolyzed nitrogen of 94.50 mg kg^−1^, available phosphorus of 21.50 mg kg^−1^, and available potassium of 240.50 mg kg^−1^. The soil’s pH (8.40) was measured according to ISO 10390:2021 [21], organic matter content (18.31 g/kg) was determined following ISO 14235:1998 [22], available phosphorus (21.50 mg kg^−1^) was assessed using ISO 11263:1994 [23], and available potassium (240.50 mg kg^−1^) was analyzed according to ISO 11260:1994 [24], while the alkali-hydrolyzable nitrogen (94.50 mg kg^−1^) was measured using the Chinese national standard GB/T 32737-2016 due to the lack of a specific ISO standard [25].

### 2.2. Microplastic Aging

Microplastics were mixed with soil at a ratio of 10% (*w*/*w*), shaken for 24 h at 180 r min^−1^, filtered through a 0.45 µm glass fiber filter, and subsequently dried at 40 °C for 6 h. The stirrer used in the experiment was a magnetic stirrer (Model: NHWY-2102, Changzhou Henglong Instrument Co., Ltd., Changzhou, China). The process of aging in soil is widely recognized as it induces physicochemical changes in microplastics, such as oxidation, surface roughening, and microcrack formation, which can significantly influence their adsorption properties [18]. Microplastics premixed with soil are referred to as treated microplastics, in contrast to pristine microplastics, which did not undergo soil treatment. This aging process mimics real environmental conditions where microplastics interact with soil matrices, thereby altering their surface functionality and adsorption behavior [19].

### 2.3. Characterization of Microplastics

To investigate the surface morphology and elemental composition of aged and pristine microplastics, approximately 10 mg of the sample was adhered to sample holders and sputter-coated with gold before observations were carried out via a scanning electron microscope (SEM, ZEISS Sigma 300, Darmstadt, Germany). SEM analysis has been extensively used to reveal surface features such as roughness, cracks, or pits induced by aging [20]. Elemental analysis was performed to assess the distribution and concentration of elements within the field of view.

Furthermore, X-ray photoelectron spectroscopy (XPS, Thermo Scientific K-Alpha, Waltham, MA, USA) was used to analyze the chemical states and concentrations of elements on the microplastic surfaces. XPS is a powerful tool for identifying oxidation-induced functional groups on microplastics, such as carbonyl or hydroxyl groups, which can enhance adsorption capacity. Additionally, functional groups were characterized using attenuated total reflection Fourier transform infrared spectroscopy (ATR-FTIR, Nicolet iS50, Thermo Fisher, Waltham, MA, USA) across wavenumbers ranging from 4000 to 400 cm^−1^ with a resolution of 0.5 cm^−1^. The identification of functional groups through FTIR has been widely documented as being essential to understanding the adsorption mechanisms [26].

### 2.4. Adsorption of BaP by the Microplastic–Soil Mixture

To evaluate the adsorption behavior of BaP by the microplastic–soil mixture, 0.1 mg of microplastics was added to 1 g of soil (mass ratio of 10%). The soil weight was based on dry weight (d.w.). Then, 20 mL of 0.01 mg/L CaCl_2_ solution was added to the mixture. BaP was introduced to achieve a concentration of 1.0 mg/L. The calcium chloride (CaCl_2_, anhydrous, purity ≥ 99.0%) used in the experiment was supplied by Shanghai Chemical Reagent Co., Ltd., Shanghai, China. At 25 °C, the samples were oscillated in a constant-temperature shaker at 180 r/min for 1440 min, simulating environmental adsorption conditions [27]. The supernatants were collected, centrifuged at 12,000 rpm for 10 min, purified, and concentrated. The detection of BaP in the supernatants was conducted via high-performance liquid chromatography (HPLC). Each treatment was conducted in triplicate for statistical accuracy.

### 2.5. Adsorption of BaP by Microplastics

#### 2.5.1. Adsorption Kinetics Experiment

BaP was dissolved in acetone to prepare a stock solution with a mass concentration of 4 g L^−1^. This stock solution was diluted to a concentration of 1 mg L^−1^, as required for the experiment, and a 0.01 mol L^−1^ CaCl_2_ solution was used as the background solution. PS, PVC, and PE (0.1 g) were weighed into separate 100 mL centrifuge tubes, followed by the addition of 20 mL of 0.01 mg L^−1^ CaCl_2_ solution and an appropriate amount of 1 mg L^−1^ BaP solution. The experimental treatments were as follows: PS+1 mg L^−1^ BaP, PVC+1 mg L^−1^ BaP, and PE+1 mg L^−1^ BaP. At 25 °C, the samples were oscillated in a constant-temperature shaker, and the centrifuge tubes were subsequently placed in a constant-temperature (25 °C) water bath shaker at 180 r min^−1^. At 5, 10, 15, 30, 60, 90, 180, 360, 720, and 1440 min, the supernatants were collected, centrifuged at 12,000 rpm for 10 min, and subsequently purified and concentrated. Detection was performed via HPLC, with each treatment conducted in triplicate.

#### 2.5.2. Adsorption Thermodynamics Experiment

PS, PVC, and PE (0.1 g) were weighed into separate 100 mL centrifuge tubes, followed by the addition of 20 mL of 0.01 mg L^−1^ CaCl_2_ solution. BaP was added to achieve concentrations of 0.2, 0.4, 0.6, 0.8, 1, 3, and 5 mg L^−1^. At 25 °C, the samples were oscillated in a constant-temperature shaker, and the centrifuge tubes were subsequently placed in a constant-temperature (25 °C) water bath shaker at 180 r min^−1^ for 1440 min. The supernatants were collected, centrifuged at 12,000 rpm for 10 min, and subsequently purified and concentrated. Detection was performed via HPLC, with each treatment conducted in triplicate.

### 2.6. Purification and Concentration of BaP and Detection

The supernatant collected after centrifugation was combined with 20 mL of dichloromethane and shaken for 30 min at 170 rpm to ensure thorough mixing. After shaking, the mixture was allowed to stand for half an hour to separate the organic and aqueous layers. The aqueous layer was subsequently aspirated, leaving behind the organic layer. Anhydrous sodium sulfate was then added to the organic layer to remove any residual moisture. The treated samples were collected into rotary evaporation flasks and repeatedly rinsed with dichloromethane before being concentrated in a rotary evaporator (40 °C water bath, 206 Pa). After evaporating the dichloromethane, 1.5 mL of acetonitrile was added, resulting in the concentration of BaP. The concentrated samples were filtered through a 0.22 μm organic membrane filter to remove the suspended particles. Detection was performed via HPLC. The chromatographic conditions were as follows: the mobile phase consisted of a mixture of acetonitrile and water at a 90:10 ratio and was introduced at a flow rate of 1 mL min^−1^. The detection wavelength was set at 264 nm, the retention time was 18 min, and the injection volume was 10 μL.

### 2.7. DFT Calculations

All density functional theory (DFT) calculations were performed with the Gaussian16 A03 software package. The geometry optimization calculations were performed via the B3LYP functional and the 6-31G* basis with the PCM solvation model for water, which included Grimme dispersion corrections (GD3BJ). Then, the singlet point energy calculations were carried out based on the B3LYP functional and a larger def2-TZVP bases with the SMD solvation model for water, which included Grimme dispersion corrections (GD3BJ). The binding energy was calculated via the following equation:

(1) $$\;E=E_{complex}-\left(E_{Polymer}+E_{Benzopyrene}\right)$$

where $E_{complex}$ represents the total energy of the complex formed by the polymer and BaP; $E_{Polymer}$ represents the total energy of the polymer on its own; and $E_{Benzopyrene}$ represents the total energy of the BaP on its own.

### 2.8. Data Analysis and Statistical Analysis

The adsorption capacity of BaP (*Q*, mg g^−1^) was calculated via the following formula:

(2) $$Q=\frac{\left(C_{0}-C_{e}\right)\times V}{m}$$

where $C_{0}$ and $C_{e}$ represent the initial concentration and equilibrium concentration of BaP in the solution, respectively (mg L^−1^); *V* represents the volume of the solution (L); and mm represents the mass of the soil (g).

Freundlich equation:

(3) $$q_{e}=K_{F}C_{e}^{1/n}$$

where $q_{e}$ is the amount of BaP per unit mass of soil at equilibrium (mg g^−1^); $C_{e}$ is the equilibrium concentration of BaP (mg L^−1^); and $K_{F}$ (mg^−1^ g L g^−1^); and n represents the empirical constants characterizing the adsorption capacity or strength of the adsorbent. When 2 < n < 10, adsorption is easy and strong; when n < 0.5, adsorption is difficult.

The equation for the linear model is as follows:

(4) $$q_{e}=K_{d}C_{e}$$

where $q_{e}$ is the amount of BaP per unit mass of soil at equilibrium (mg g^−1^) and $K_{d}$ is the partition coefficient of BaP (L mg^−1^) between the adsorbent and the aqueous solution.

The adsorption kinetics characteristics were fitted by pseudo-first-order and pseudo-second-order kinetic equations as follows:

Pseudo-first-order kinetic equation:

(5) $$q_{t}=Q_{e,1}\left(1-e^{-k_{1}t}\right)$$

Pseudo-second-order kinetic equation:

(6) $$q_{t}=\frac{Q_{e,2}^{2}k_{2}t}{1+Q_{e,2}k_{2}t}$$

where $q_{t}$ is the adsorption amount of BaP at any time *t* (mg g^−1^); $Q_{e,1}$ and $Q_{e,2}$ represent the adsorption equilibrium amounts (mg g^−1^); and $k_{1}$ and $k_{2}$ represent the adsorption rate constants (g mg^−1^ min^−1^).

The pseudo-first-order kinetic equation typically describes the adsorption process at low concentrations, representing a proportional relationship between the adsorption rate and the number of active sites remaining on the adsorbent surface. The pseudo-second-order kinetic equation assumes that the adsorption process is limited by the active sites on the adsorbent surface and that the adsorption capacity is related to the coverage of the adsorbate on the adsorbent surface. This is usually applicable when the surface site saturation of the adsorbent is relatively high.

The intraparticle diffusion equation is as follows:

(7) $$q_{t}=K_{p}t^{1/2}$$

where $q_{t}$ (mg g^−1^) is the amount of BaP adsorbed on the microplastics at time *t* and $K_{p}$ (g mg^−1^ h^−0.5^) is the diffusion rate constant of BaP inside the particles.

The relative distributions of BaP in microplastics and soil were determined by calculating the relative partition coefficients of BaP in single systems (soil or microplastics) and mixed systems (microplastics–soil).

(8) $$K_{d}/K_{MP}=Q_{e}/C_{e}$$

(9) $$K_{MP}=\left(K_{total}-K_{d}\right)/\;f_{MP}$$

where $Q_{e}$ (mg g^−1^) and $C_{e}$ (mg L^−1^) represent the amount of BaP adsorbed on the adsorbent and the concentration in solution at equilibrium, respectively. $K_{total}$ represents the total partition coefficient of BaP in soil with added microplastics, and $f_{MP}$ (%) represents the content of microplastics in the soil. MP refers to PE, PS, and PVC. Data processing was performed via Origin Pro 2021 software to fit the adsorption and desorption of BaP in different characteristic soils. A statistical analysis of the data was conducted via SPSS 21.0 and Microsoft Excel 2017. Multiple comparisons were made via the least significant difference (LSD) method, with the significance level set at *p* < 0.05.

## 3. Results and Discussion

### 3.1. Adsorption of BaP Microplastics

**Figure 1:**
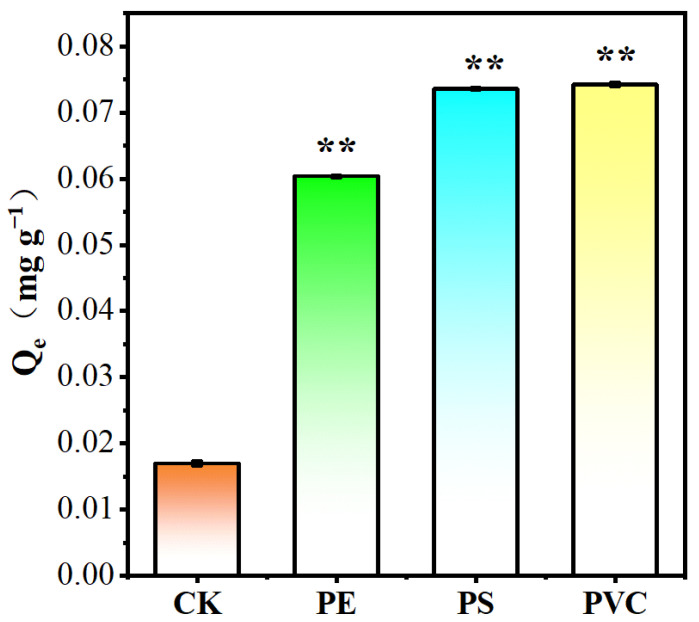
CK shows the adsorption of BaP by soil, and the others show the adsorption of BaP by 10% microplastics + soil, ** Significance: *p* < 0.01.

**Figure 2:**
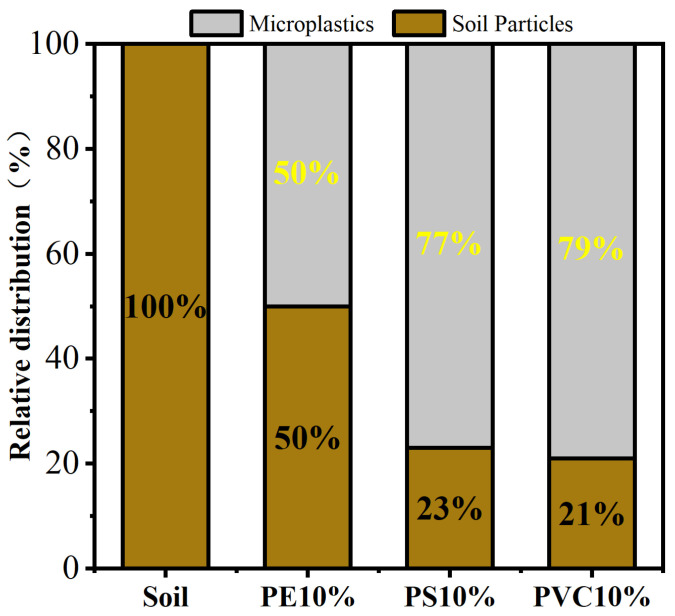
The relative distribution of BaP between soil and PE, PS, and PVC microplastics at addition rates of 10%.

To investigate the adsorption of BaP by soil, microplastics were mixed with soil at a ratio of 0.1 g of microplastics to 1 g of soil, as shown in Figure 1. The results indicated that with an initial BaP concentration of 1 mg L⁻^1^, the amounts of BaP adsorbed by the soil with added PE, PS, and PVC were 0.060 mg g⁻^1^, 0.072 mg g⁻^1^, and 0.075 mg g⁻^1^, respectively. Compared with adsorption by soil alone (0.017 mg g⁻^1^), the addition of microplastics enhanced BaP adsorption by 2.53 times (PE), 3.50 times (PS), and 3.69 times (PVC). To better elucidate the role of microplastics in the adsorption of BaP within the soil–microplastic system, we further analyzed the distribution of BaP between the soil and microplastic particles, as shown in Figure 2. When 10% microplastics were added to the soil, the distribution ratios of BaP in the soil and PE microplastics were 50% each. In contrast, the distributions of BaP on PS microplastics and the soil were 77% and 23%, respectively, while the distributions of BaP on PVC microplastics and the soil were 79% and 21%, respectively. These results indicate that BaP preferentially adheres to microplastics over soil, with the affinity order being PVC > PS > PE, which is consistent with the adsorption outcomes.

In single-microplastic systems, the adsorption capacity of BaP followed the trend PVC > PS > PE. The same trend was observed in the soil–microplastics systems, indicating that microplastics dominated BaP adsorption in the mixed systems, which is consistent with previous studies [14]. Some studies have reported that the adsorption of pesticides in horticultural soil on PE films was 1–2 orders of magnitude greater than that on soil [28]26. For example, the adsorption of chlorpyrifos on PE was 2284 μg g⁻^1^, whereas it was only 32 μg g⁻^1^ on soil. Microplastics in soil have a strong phenanthrene adsorption capacity, significantly affecting phenanthrene’s adsorption and distribution in soil [18].

These studies demonstrate that microplastics can enhance the adsorption of organic pollutants in soil. Compared with soil organic matter, microplastics may serve as more significant sinks for organic pollutants in soil.

### 3.2. Characterization of Pristine and Soil-Treated Microplastics

**Figure 3:**
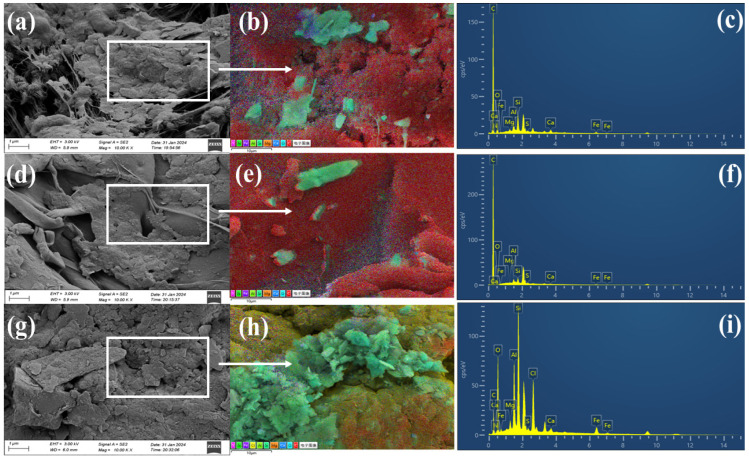
SEM-EDS images of microplastics adsorbing BaP after soil pre-treatment (PE, PVC and PS), PE (**a**–**c**), PS (**d**–**f**) and (**g**–**i**) with magnification of 10,000× for image. Elemental composition (via EDS) is shown as insets in each subpanel. PE (**b**,**c**), PS (**e**,**f**) and PVC (**h**,**i**).

The three types of microplastics exhibit distinct morphologies. Pristine microplastics appear relatively smooth, with irregular elliptical and some spherical particles. Under 10,000× magnification, the fibrous structure of PE is clearly visible in Figure S1. After being treated with soil, the surface morphology and elemental composition of the microplastics significantly changed, as depicted in Figure 3a–i. The surface of the PE microplastics became rougher and more irregular, indicating a more pronounced fibrous structure after treatment. PS microplastics develop additional pits and cracks, as shown in Figure 3d–f, and the gaps between PVC particles become more distinct, as shown in Figure 3g–i. The surfaces of PE and PS are primarily composed of carbon (over 99%), with minimal oxygen, whereas PVC contains 89% carbon and 10% chlorine. Trace amounts of oxygen have also been detected on pristine microplastics in prior studies [17]. An EDS analysis revealed that these layers consisted primarily of carbon (80.56%), oxygen (11.65%), silicon (2.94%), calcium (1.09%), aluminum (1.37%), and iron (1.75%), as detailed in Table 1. These mineral elements likely originate from tidal soils, which are known for their rich organic content and high water retention capabilities and are predominantly composed of hydrous mica. PVC shows the most significant elemental changes posttreatment. The analysis revealed that its surface composition consists of carbon (32.79%), oxygen (28.42%), chlorine (8.83%), silicon (14.58%), aluminum (6.8%), iron (6.23%), and calcium (0.85%). The notable reduction in carbon content is attributed to the adherence of minerals and organics from the tidal soil, which cover part of the original PVC material. Owing to surface oxidation, this organic mineral layer leads to a significant reduction in carbon and an increase in oxygen content. Changes on the PS surface primarily involve an increase in oxygen content, with the content of metallic elements remaining below 1%. These findings suggest that soil pretreatment leads to microplastic oxidation, altering their surface functional groups. These changes are likely due to microbial degradation over time. Microplastics in natural environments become more brittle over time [29]. Moreover, pretreated microplastics develop more oxygen-containing functional groups on their surfaces. The increase in the number of C–O bonds on all three types of microplastics, especially on PE and PVC, indicates a greater degree of oxidation. Conversely, the surface of PS remains relatively stable, possibly due to shorter soil pretreatment durations [30].

**Figure 4:**
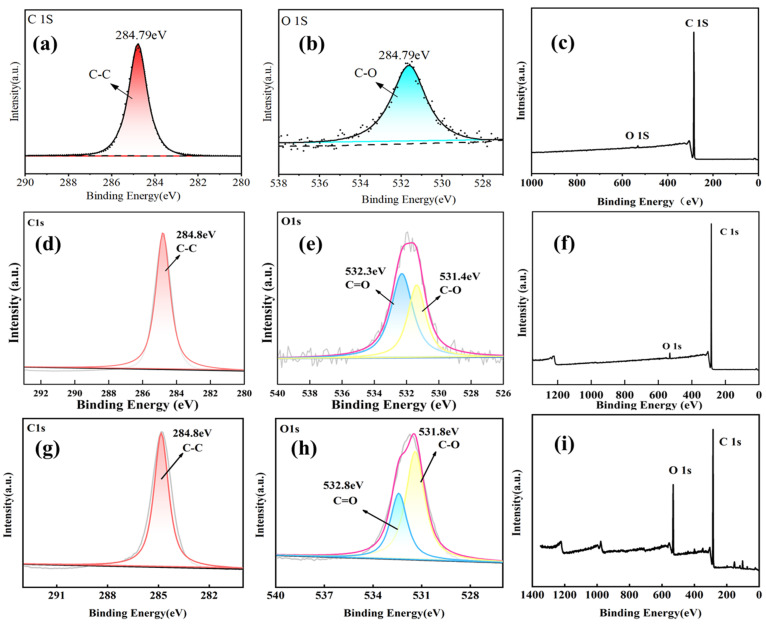
XPS spectra of PE microplastics before and after sorption; high-resolution XPS spectra of C 1s (**c**) and O 1s (**d**) regions of PE microplastics. PE (**a**–**c**), PE+BaP (**d**–**f**), and PE+Soil+BaP (**g**–**i**).

An XPS analysis of PE, PS, and PVC microplastics under different treatment conditions revealed changes in their surface chemistry, as shown in Figure 4, Figures S3 and S4. After BaP adsorption, peaks for C=O and C-O bonds appear in the O 1s spectra of all three microplastics in Figure 4b, Figures S3f and S4f, indicating the introduction of oxygen-containing functional groups by BaP [30]. Changes in the peaks in the C 1s spectra further indicate that BaP adsorption alters the chemical properties of the microplastic surfaces through chemical interactions, which is especially evident in the spectra of PS and PVC, where increased π–π interactions suggest interactions between BaP molecules and microplastic surfaces [31]. The π–π interactions are considered the primary adsorption mechanism by which PS microplastics adsorb aromatic compounds [32]. After soil pretreatment and BaP adsorption, the peak attributed to the aromatic carbon of PS shifted from 291.1 to 291.6 eV, and the peak attributed to the aromatic carbon on the surface of PVC was located at 293.3 eV (Figures S3g and S4i). This phenomenon indicates that the π–π interaction is enhanced after soil pretreatment [26].

Figure S5 shows the infrared spectral features of the PE, PS, and PVC microplastics under different treatment conditions. The vibrational bands in the ranges of 2906–2851 cm^−1^, 3081–3026 cm^−1^, and 3082–3002 cm^−1^ include typical C–H stretching peaks [33], corresponding to the fundamental structures of the polymers. The pristine PE microplastics show C=C stretching peaks in the range of 1680–1600 cm^−1^, which increase in intensity after BaP adsorption. New peaks, such as those at 1027 cm^−1^ indicating Si–O stretching [18], are observed for the surface of the PE microplastics after soil pretreatment, which is consistent with the EDS elemental analysis results. Additionally, the EDS and FTIR analyses suggest that the coverings observed in the SEM images of real environmental microplastic samples might consist of several elements (such as O, Fe, and Si), indicating the attachment of clay minerals (such as iron oxides and silicon oxides) on the surfaces of the microplastics [34]. The intensities of the vibrational peaks at 1740 cm^−1^ and 1147 cm^−1^ for the PS microplastics subjected to different treatments vary, indicating weak C–O and C–O–C stretching after BaP adsorption and soil pretreatment [35]. In the PS microplastics that adsorbed BaP, a new peak at 1731.79 cm^−1^ representing C=O vibrations was observed, and considering EDS analysis, the oxygen content on the PVC microplastics increased to 28.42% after soil pretreatment. The adsorption of BaP significantly enhances the intensity of the infrared spectral peaks of all three types of microplastics, indicating that interactions between BaP and microplastic surfaces alter their chemical properties. After soil treatment, the infrared spectra of the microplastics show new absorption peaks and changes in intensity, indicating that soil pretreatment introduces new functional groups or increases the number of active surface sites, increasing the adsorption capacity for BaP.

**Figure 5:**
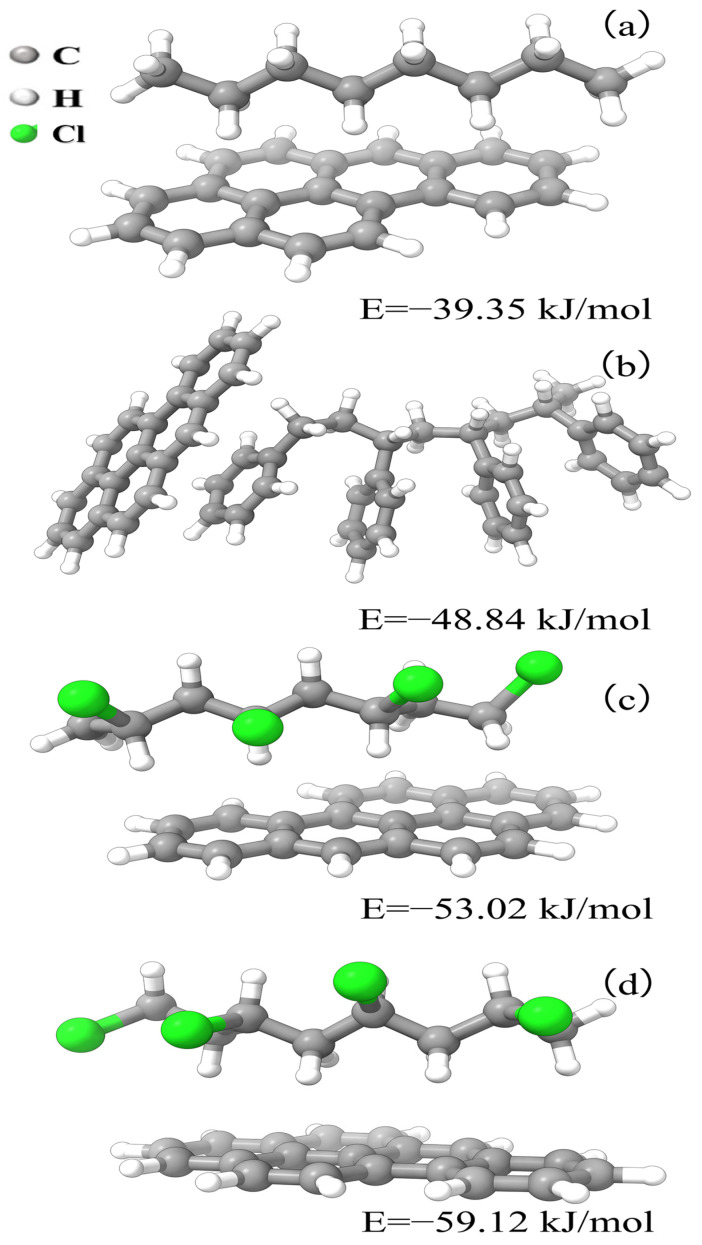
Molecular Models and binding energies of different microplastics (PE, PS, PVC) with BaP, PE (**a**), PS (**b**), PVC (**c**,**d**).

Figure 5 displays the molecular models and binding energies of BaP with the three types of microplastics (PE, PS, and PVC). The binding energy of PE with BaP is −39.35 kJ/mol; Figure 5a indicates a weak interaction, primarily through van der Waals forces, which limits the adsorption capacity of BaP under environmental conditions. In combination with Figure 3, it can be seen that the microplastics after soil treatment also bind BaP through van der Waals forces. The binding energy of PS with BaP is −48.84 kJ/mol. Figure 5b shows that PS, with its aromatic ring structure, can form stronger π–π interactions with BaP, resulting in a stronger adsorption capacity. Figure S3 shows that soil pretreatment with microplastics enhances π–π interactions, the microplastics pretreated with soil provided more sorption binding sites for BaP, thereby facilitating the attachment of BaP to the microplastics [36]. Moreover, the binding energy of PVC with BaP is −53.02 kJ/mol. Figure 5c combined with Figure S4 shows that the polar chlorine atoms in PVC enhance van der Waals and dipole–dipole interactions. After soil pretreatment, the microplastics formed stronger π–π interactions with BaP, with a binding energy of −59.16 kJ/mol, and Figure 5d shows the strongest interaction. After soil pretreatment, the BaP adsorption mechanism of the PVC microplastics shifts from dipole–dipole interactions to π–π interactions, and research has shown that after aging in soil, the adsorption of organic pollutants by microplastics shifts from hydrophobic and π–π interactions to hydrogen bonding and π–π interactions [19].

### 3.3. Adsorption of Benzo[a]pyrene by Microplastics

**Figure 6:**
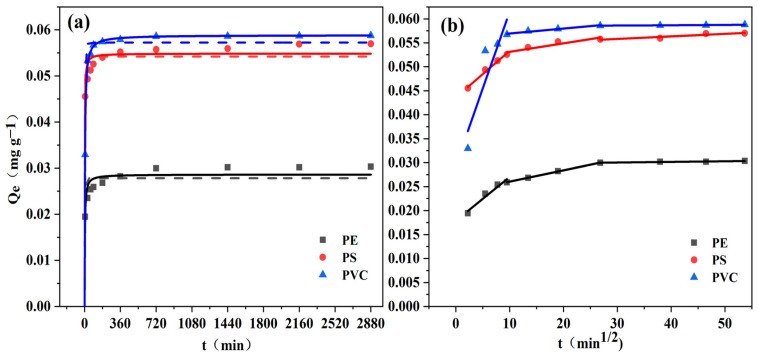
Adsorption kinetics of BaP on PE, PS, and PVC microplastics. The initial concentration of BaP was 1 mg L^−1^ and the background solution was 0.01 mol L^−1^ CaCl_2_. (**a**) The dashed and solid lines in the figure represent pseudo primary and pseudo secondary kinetic model fits, respectively, and (**b**) the solid line in the figure represents the intra-particle diffusion model fit.

The adsorption kinetics of BaP on the three types of microplastics are shown in Figure 6a. In the initial stage, the adsorption rates of BaP on the PS and PVC microplastics were significantly higher than those on the PE microplastics. The times required for the three microplastics to adsorb 90% BaP were approximately 180 min, 60 min, and 30 min. In the initial adsorption stage, BaP rapidly attaches to the soil or soil microplastics through mass transfer [37]. The pseudo-second-order kinetic model accurately described the adsorption kinetics of BaP on PE, PS, and PVC microplastics (R^2^ > 0.95), as shown in Table 2, with PVC showing the best fit (0.999). The equilibrium adsorption amounts ($Q_{e}$) and adsorption rates ($K_{2}$) of BaP on the three microplastics differed significantly (*p* < 0.01). PS exhibited the highest adsorption rate, reaching apparent equilibrium at 360 min, with $Q_{e}$ reaching 0.055 mg g⁻^1^. PVC had the lowest adsorption rate but the largest adsorption amount, reaching apparent equilibrium at 1440 min, with $Q_{e}$ reaching 0.059 mg g⁻^1^. PE had the smallest adsorption amount, reaching apparent equilibrium at 720 min, with a $Q_{e}$ of only 0.029 mg g⁻^1^.The hydrophobicity of microplastics is crucial for the adsorption of nonpolar organic pollutants such as BaP. Microplastics with greater hydrophobicity may more effectively adsorb BaP, as BaP tends to migrate from the aqueous phase to more hydrophobic surfaces [38].

The adsorption kinetics data were further fitted via the intraparticle diffusion model. As shown in Figure 6b, the adsorption kinetics of BaP on the three microplastics could be better described by the intraparticle diffusion model, which divides the adsorption process into three stages: surface diffusion, external diffusion, and micropore diffusion. The intercepts of the fitting curves for the three microplastics were all nonzero, as shown in Table S1. This finding indicates that intraparticle diffusion was not the sole rate-limiting step and that the adsorption rates of BaP on the three microplastics were controlled by all three processes [39,40].

Previous studies have revealed that the adsorption rate of BaP on PS significantly exceeds that on PE [41]. The pseudo-second-order kinetic model has been widely applied to explain the adsorption rates of organic pollutants on various microplastics. For example, researchers have investigated the adsorption behavior of phenanthrene on PE and biodegradable microplastics and report that the adsorption processes perfectly fits the pseudo-second-order kinetic model (R^2^ = 1) [42]. The adsorption processes of tetrabromobisphenol A on PE, PS, and PVC microplastics were analyzed, revealing that these processes fit the pseudo-second-order kinetic model with R^2^ values exceeding 0.95 [43]. Similarly, the adsorption kinetics of pyrene on PE, PS, and PVC microplastics highly match the pseudo-second-order model (R^2^ > 0.99). In addition, the adsorption equilibrium time for sulfamethoxazole on six different microplastics (PE, PP, PS, PVC, PET, and PA) is approximately 16 h, and the external transfer rate is the limiting step in the adsorption process [42].

The adsorption isotherms of BaP on the three types of microplastics are shown in Figure S6. The isotherms were fitted via linear and Freundlich models, with the fitting parameters listed in Table S2. On the basis of the correlation coefficient (R^2^), the Freundlich model better described the adsorption isotherms of BaP on all three microplastics (R^2^ > 0.96). This finding indicates that the Freundlich model more accurately captures the adsorption behavior of BaP on microplastic surfaces. This result suggests that hydrophobic interactions are likely the primary mechanism driving BaP adsorption and that multilayer adsorption may also play a role, which is consistent with previous studies on the adsorption of highly hydrophobic pollutants on microplastics through partitioning [44].

Further examination of the Freundlich model parameters, particularly the deviation of the n values, revealed the nonlinear nature of BaP adsorption on microplastics. Notably, the n value of PVC significantly deviated from 1, indicating a pronounced nonlinear adsorption behavior, which is potentially related to the unique surface properties and structure of PVC. π–π interactions are considered the primary adsorption mechanism for aromatic compounds on microplastics [35]. In contrast, PE and PS exhibited less pronounced nonlinear adsorption characteristics, with n values closer to 1, indicating a more linear adsorption process.

The trend observed for the linear partition coefficients ($K_{d}$) of BaP on the three microplastics was PVC (4.319 L g⁻^1^) > PS (3.784 L g⁻^1^) > PE (2.564 L g⁻^1^), as shown in Table S2. The partition coefficient of PVC was nearly double that of PE, indicating a significantly higher adsorption capacity for BaP. Research has demonstrated that the partitioning capacity of PVC exceeds that of other microplastic varieties [41]. Notably, PE showed the highest linear fitting accuracy, which is related to its physicochemical properties. PE, as a rubbery polymer, has flexible polymer chains that facilitate the diffusion of organic pollutants within its structure, leading to more linear adsorption behavior for BaP. Conversely, PS and PVC are glassy polymers at room temperature, where hydrophobic organic pollutants are adsorbed into the nanopores characteristic of glassy polymers, usually resulting in a nonlinear process [43].

## 4. Conclusions

This study thoroughly examined the BaP adsorption behaviors of various microplastics (PE, PS, and PVC) and their interactions with soil systems. DFT calculations revealed that the BaP binding energy of soil pretreated with PVC was lower (−59.16 kJ/mol), indicating more stable adsorption through π–π interactions. Additionally, the XPS results revealed changes in the chemical states of the microplastic surfaces after soil treatment, particularly an increase in the content of oxygen and functional groups on PVC, which facilitated chemical adsorption with BaP. Compared with soil alone, microplastics significantly increased the adsorption capacity for BaP, establishing them as primary sinks for such organic pollutants. However, despite these findings, the current study has certain limitations. For example, the focus was primarily on the adsorption behavior of BaP in microplastics and soil–microplastic systems under laboratory conditions, which may not fully replicate real-world environmental scenarios. Additionally, the effects of dynamic environmental factors, such as temperature fluctuations, microbial activity, and competitive adsorption with other pollutants, remain insufficiently explored. The presence of microplastics in the soil not only increased the overall BaP adsorption but also altered its distribution within the soil–microplastic system, potentially affecting the mobility and bioavailability of BaP in the environment. This research provides valuable insights into the environmental dynamics of microplastics. Considering that pollutants often persist at low concentrations in real-world environments, future studies should assess the impact of the long-term, low-concentration coexistence of BaP and microplastics on crops and in soil ecosystems. For future research, it is recommended to focus on the long-term, low-concentration coexistence of BaP and microplastics, particularly their impacts on crops and soil ecosystems under field conditions.

## Figures and Tables

**Table 1:** Surface composition of raw microplastics and soil-treated EDS elements.

Microplastics	EDS Elemental Surface Composition (%)						
	C	O	Cl	AI	Si	Fe	Ca
PE	99.25	0.40	—	—	—	—	—
PE+BaP	98.86	0.71	—	—	—	—	—
Soil Pretreatment PE+BaP	80.56	11.65	—	1.37	2.94	1.75	1.09
PS	99.81	—	—	—	—	—	—
PS+BaP	99.18	0.42	—	—	—	—	—
Soil Pretreatment PS+BaP	91.17	5.76	—	0.71	1.22	0.57	0.09
PVC	88.73	0.63	10.25	—	—	—	—
PVC+BaP	90.01	0.97	8.60	—	—	—	—
Soil Pretreatment PVC+BaP	32.79	28.42	8.83	6.8	14.58	6.23	0.85

**Table 2:** Fitted parameters for adsorption kinetics of BaP on different microplastics.

Microplastics	Pseudo-First-Order Kinetic Equation			Pseudo-Second-Order Kinetic Equation		
	Q_e_ (mg g^−1^)	K_1_ (g mg^−1^ min^−1^)	R^2^	Q_e_ (mg g^−1^)	K_2_ (g mg^−1^ min^−1^)	R^2^
PE	0.028 ± 0.001	0.238 ± 0.058	0.927	0.029 ± 0.001	11.692 ± 3.230	0.964
PS	0.054 ± 0.001	0.366 ± 0.060	0.976	0.055 ± 0.001	15.256 ± 3.761	0.986
PVC	0.057 ± 0.001	0.169 ± 0.015	0.990	0.059 ± 0.000	4.430 ± 0.151	0.999

## Data Availability

The data that has been used is confidential.

## Supplementary Materials

The following supporting information can be downloaded at: https://www.mdpi.com/article/10.3390/toxics12120922/s1, Figure S1: SEM-EDS images of microplastics (PE, PVC and PS) PE (a–c), PS (d–f) and PVC (g–i) with magnification of 10,000× for image.; Figure S2: SEM-EDS images of microplastics adsorbing BaP (PE, PVC and PS), PE (a–c), PS (d–f) and PVC (g–i) with magnification of 10,000× for image; Figure S3: XPS spectra of PS microplastics before and after sorption, high resolution XPS spectra of C 1s (c) and O 1s (d) regions of PS microplastics. PS (a–c), PS+BaP (d–f), and PS+Soil+BaP (g–i); Figure S4: XPS spectra of PVC microplastics before and after sorption, high resolution XPS spectra of C 1s (c) and O 1s (d) regions of PVC microplastics. PVC (a–c), PVC+BaP (d–f), and PVC+Soil+BaP (g–i); Figure S5: FTIR spectra of microplastics (PE, PS and PVC) before and after sorption; Figure S6: Adsorption isotherms of BaP on PE, PS, and PVC microplastics. The initial concentration of BaP was 1 mg L^−1^ and the background solution was 0.01 mol L^−1^ CaCl_2_. The realized and dashed lines represent the adsorption isotherms using Freundlich model and linear fitting, respectively; Table S1: Intraparticle diffusion fitting parameters for BaP in different microplastics; Table S2 Regression parameters of BaP adsorption isotherms on three microplastics fitted using linear and Freundlich models.
