# Supplementary material for: The Adsorption Process and Mechanism of Benzo[a]pyrene in Agricultural Soil Mediated by Microplastics
Source: Toxics. 2024 Dec 19;12(12):922. doi: 10.3390/toxics12120922 (PMC11728619; doi:10.3390/toxics12120922)
Supplement: Supplementary file 1 [file toxics-12-00922-s001.zip › toxics-3309763-supplementary.pdf]

Supplementary Information

For

The adsorption process and mechanism of benzo[a]pyrene in agricultural soil mediated by microplastics

Zhengyi Zhu [a,b,1], Lijuan Sun [a,1], Qin Qin [a,c] , Yafei Sun [a], Shiyan Yang [a],

Jun Wang [a], Yang Yang [a,b], Guangkuo Gao [a], Yong Xue [a,b,c*]

a. Eco-Environment Protection Research Institute, Shanghai Academy of Agricultural Sciences, Shanghai 201403, China;

b. College of Marine Ecology and Environment, Shanghai Ocean University, Shanghai 201306, China;

c. Key Laboratory of Low-carbon Green Agriculture in Southeastern China, Ministry of Agriculture and Rural Affairs, Shanghai 201403, China)

* Corresponding author at: Shanghai Environmental Protection Monitoring Station of Agriculture, Shanghai 201403, China. E-mail address: exueyong@163.com (Y. Xue).

1 Zhengyi Zhu and Lijuan Sun contributed equally to this work.

The purification, concentration, and detection of benzo[a]pyrene（BaP）

The supernatant collected after centrifugation was combined with 20 mL of dichloromethane and shaken for 30 minutes at 170 rpm to ensure thorough mixing. After shaking, the mixture was allowed to stand for 30 minutes to separate the organic and aqueous layers. The aqueous layer was carefully removed, and the organic layer was retained. Anhydrous sodium sulfate was added to the organic layer to remove residual water. The treated sample was transferred to a rotary evaporation flask, and the evaporation flask was rinsed multiple times with dichloromethane to ensure complete sample collection. Dichloromethane was evaporated using a rotary evaporator (40°C water bath, 206 Pa). Subsequently, 1.5 mL of acetonitrile was added to concentrate the BaP solution. The concentrated sample was filtered through a 0.22 μm organic membrane to remove suspended particles. The final analysis was conducted using high-performance liquid chromatography (HPLC).

The HPLC analysis was performed under the following conditions: the mobile phase consisted of acetonitrile and water at a 90:10 ratio, with a flow rate of 1 mL/min. The detection wavelength was set to 264 nm, and the retention time was 18 minutes. The injection volume was 10 μL, and a C18 column was used for separation.

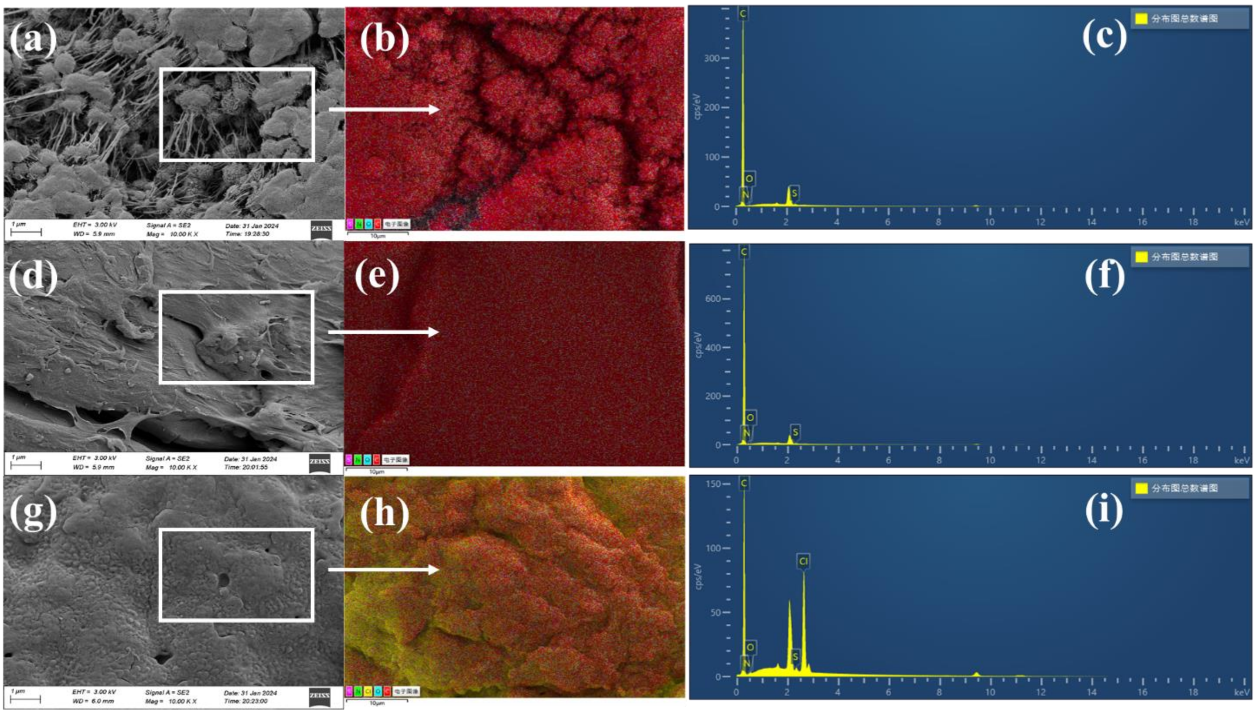

Figure S1. SEM-EDS images of microplastics (PE,　PVC and PS) PE（a-c）、PS（d-f）and PVC(g-i) with magnification of 10,000× for image.

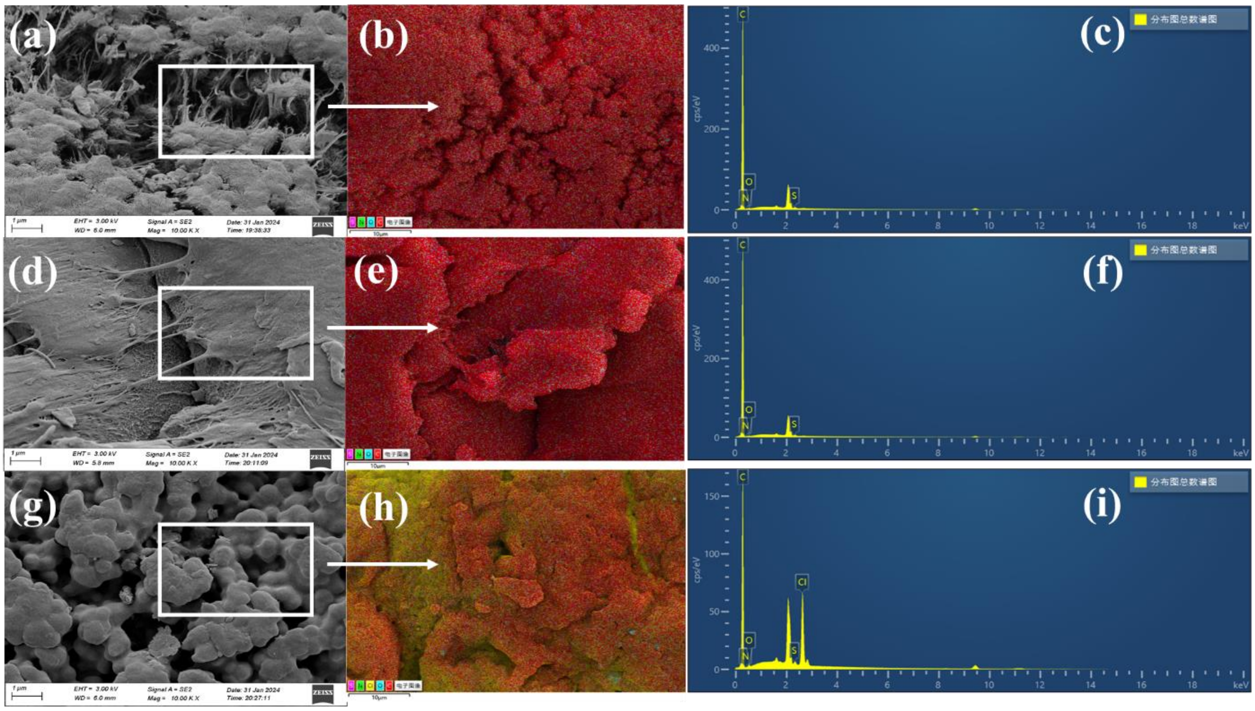

Figure S2. SEM-EDS images of microplastics adsorbing BaP (PE,　PVC and PS)，PE（a-c）、PS（d-f）and PVC(g-i) with magnification of 10,000× for image.

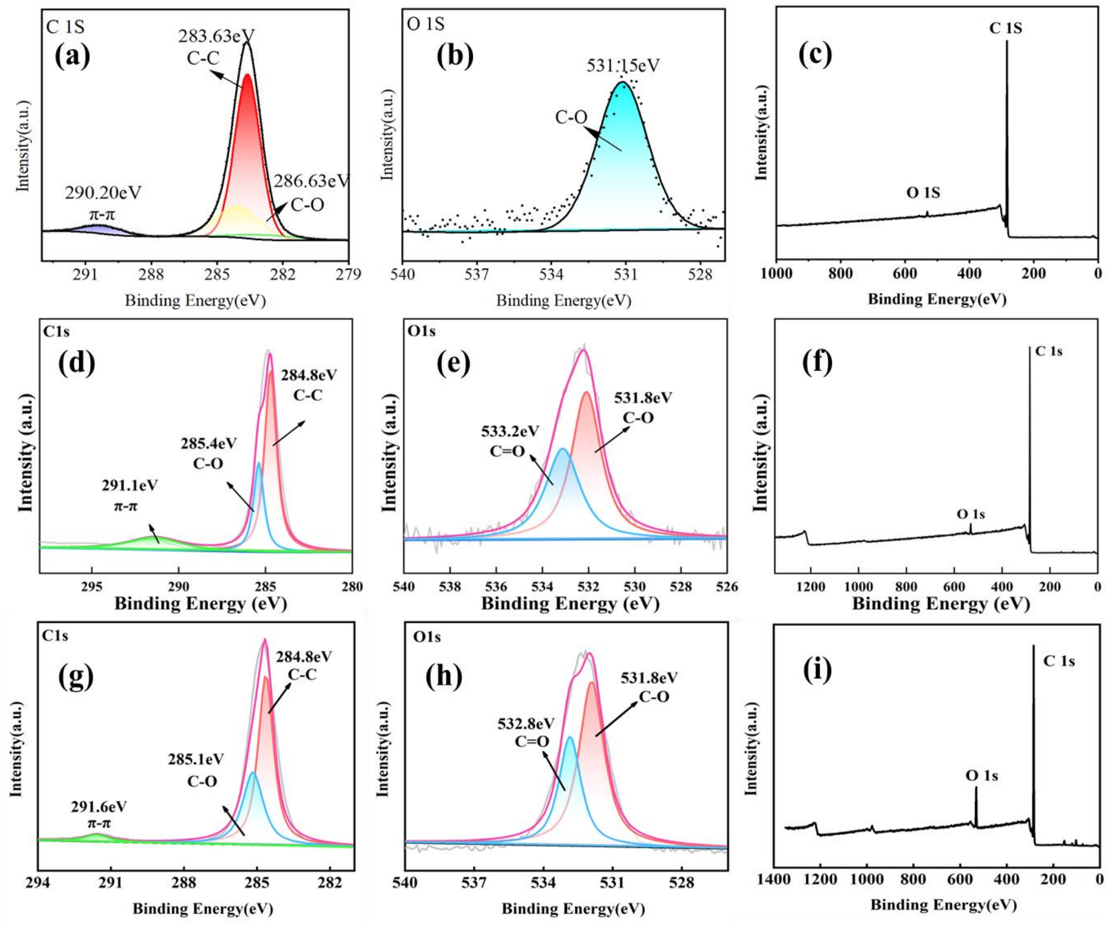

Figure S1. XPS spectra of PS microplastics before and after sorption, high resolution XPS spectra of C 1s (c) and O 1s (d) regions of PS microplastics.PS（a-b-c）, PS+BaP （d-e-f）, and PS+Soil+BaP （g-h-i）.

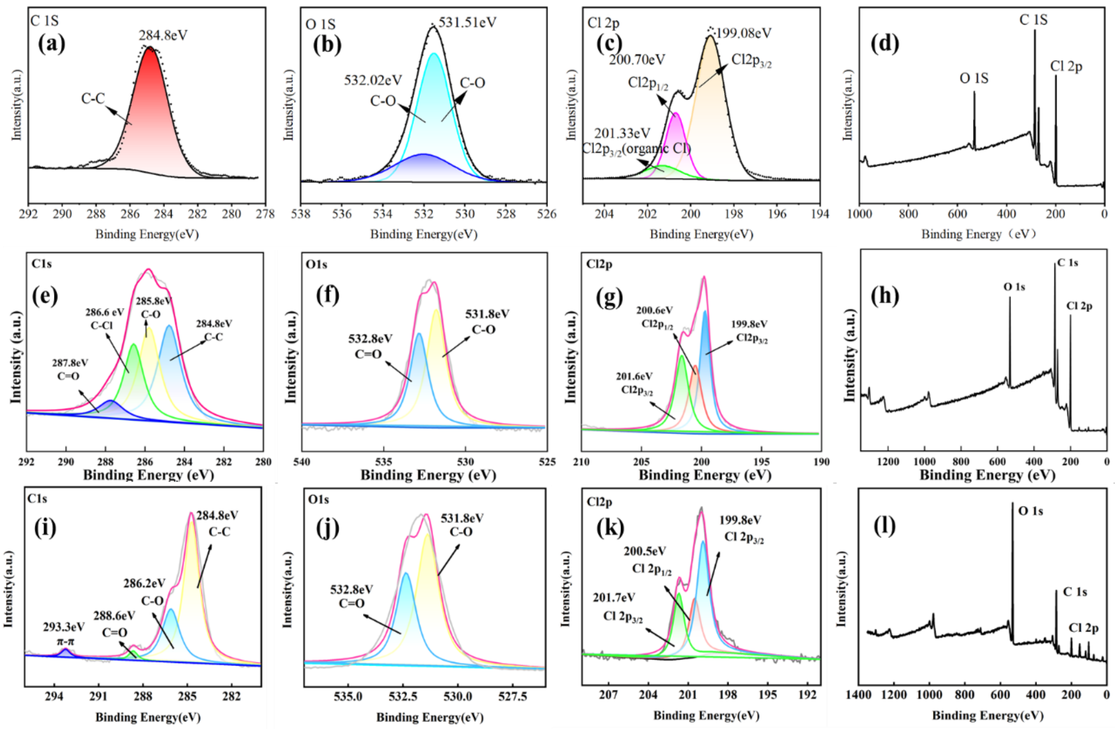

Figure S2. XPS spectra of PVC microplastics before and after sorption, high resolution XPS spectra of C 1s (c) and O 1s (d) regions of PVC microplastics. PVC（a-b-c）, PVC+BaP （d-e-f）, and PVC+Soil+BaP （g-h-i）.

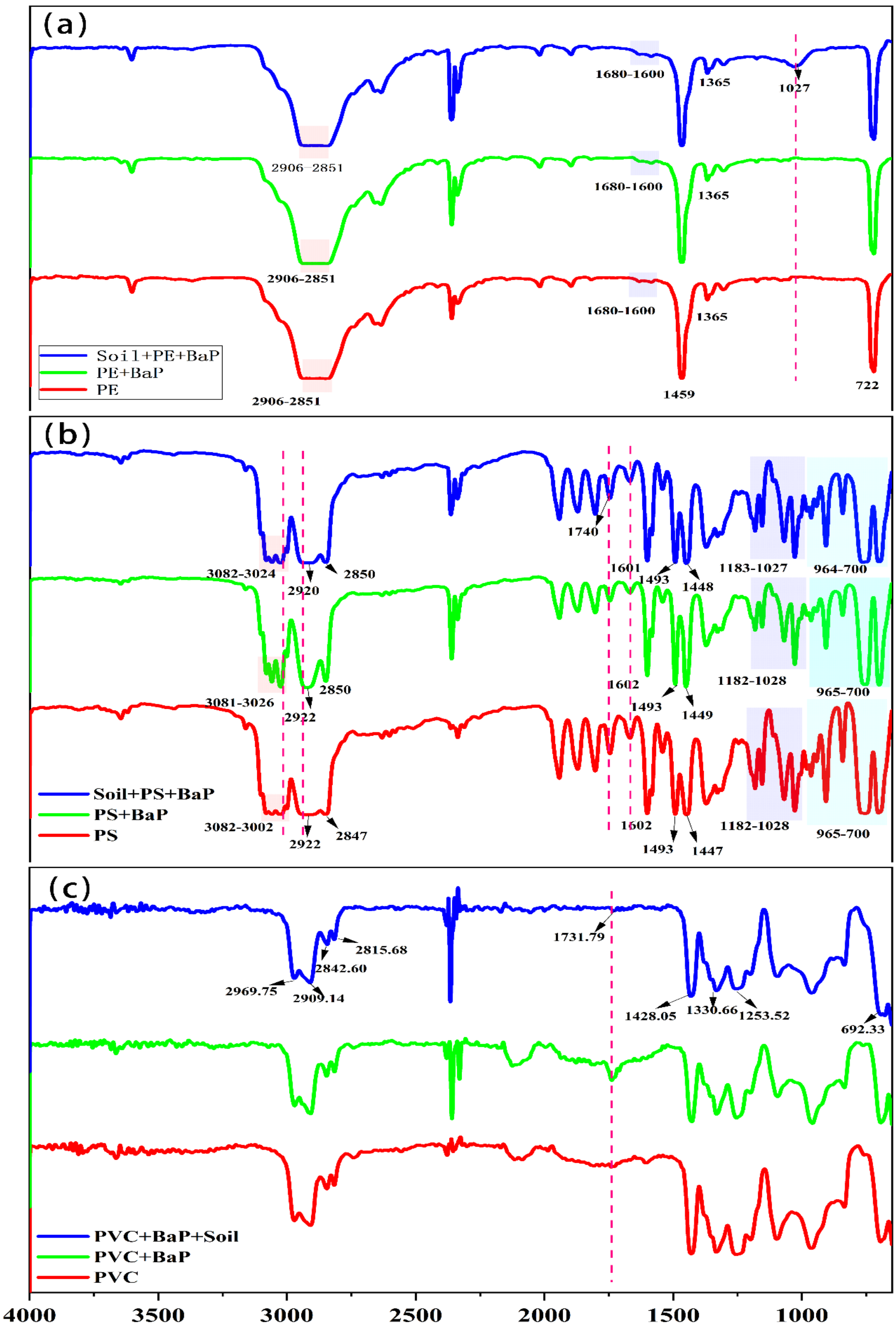

Figure S3. FTIR spectra of microplastics(PE、PS and PVC) before and after sorption.

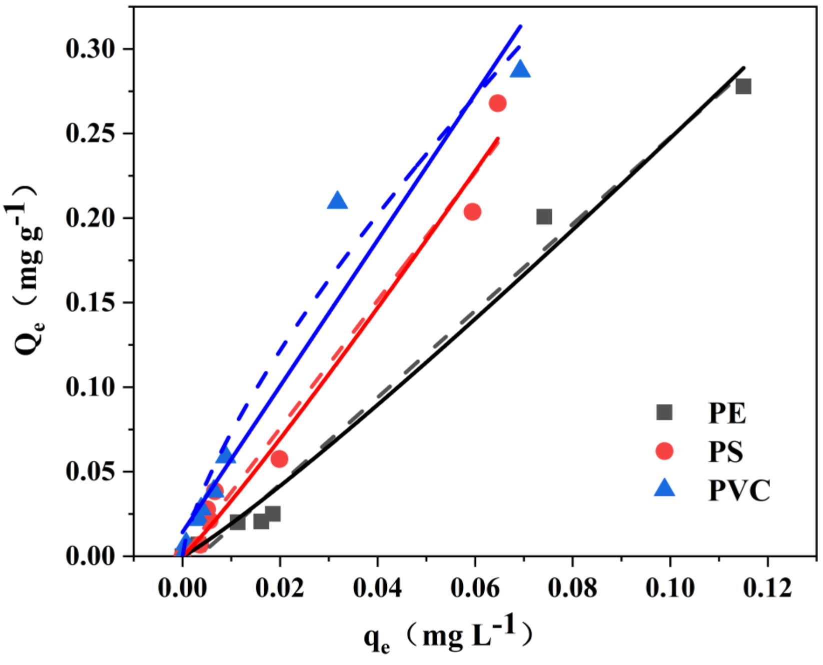

Figure S6. Adsorption isotherms of BaP on PE, PS, and PVC microplastics. The initial concentration of BaP was 1 mg L^{-1} and the background solution was 0.01 mol L^{-1} $CaCl_2$. The realized and dashed lines represent the adsorption isotherms using Freundlich model and linear fitting, respectively.

Table S1. Intraparticle diffusion fitting parameters for BaP in different microplastics

Microplastics	K_1 g mg^{-1} $h^{-0.5}$ (*10^4)	C mg g^{-1}	R^2	K_2 g mg^{-1} $h^{-0.5}$	C mg g^{-1}	R^2	K_3 g mg^{-1} $h^{-0.5}$	C mg g^{-1}	R^2
PE	9.13±1.45	0.018±0.001	0.928	2.35±0.05	0.024±0.000	0.998	0.13±0.03	0.030±0.000	0.861
PS	9.72±0.74	0.044±0.001	0.983	1.77±0.45	0.051±0.001	0.826	0.52±0.14	0.054±0.001	0.810
PVC	32.20±10.40	0.030±0.007	0.740	1.03±0.14	0.056±0.000	0.945	0.07±0.01	0.058±0.00	0.892

Table S2. Regression parameters of BaP adsorption isotherms on three microplastics fitted using linear and Freundlich models

Microplastics	Linear Model		Freundlich Model		
	K_d（L g^{-1}）	R^2	K_F	n	R^2
PE	2.564±0.127	0.985	3.171±0.856	0.902±0.093	0.984
PS	3.784±0.236	0.973	4.779±1.943	0.924±0.121	0.975
PVC	4.319±0.429	0.935	2.139±0.518	1.364±0.145	0.968
